# MambaGCN-SMI: Protocol state machine inference framework based on Mamba and GCN

**DOI:** 10.1371/journal.pone.0354921

**Published:** 2026-08-20

**Authors:** Junkang Ren, Qing Li, Ruijuan Chu, Yifan Chen

**Affiliations:** Department of Information System Engineering, PLA Information Engineering University, Zhengzhou, Henan, China; ICFAI Foundation for Higher Education Faculty of Science and Technology, INDIA

## Abstract

To address the limitations of existing non-interactive protocol state machine inference methods in feature representation capability, inter-stage information fusion, and adaptive simplification strategies, this paper proposes the MambaGCN-SMI inference framework. The method employs a dual-level Mamba encoder to extract deep semantic features from protocol interactions, constructs an initial directed state graph based on clustering and state transition conditional entropy, and utilizes a Graph Convolutional Network to learn node embeddings, enabling data-driven adaptive state merging and simplification. Experiments on a multi-protocol dataset encompassing TCP, HTTP, SMTP, Modbus, and DNP3 demonstrate that the framework outperforms mainstream methods in both state machine structure recovery and behavioral prediction. It achieves an average prediction accuracy of 95.7% and a behavior coverage of 97.5%. For structural recovery, the node and edge F1-scores reach 0.939 and 0.915, respectively. Ablation studies confirm that both the Mamba encoder and the Graph Convolutional Network module contribute significantly to the performance improvement. By integrating selective state space modeling with graph structure learning, this research achieves high-precision, adaptive inference of private protocol state machines, offering an effective deep learning solution for protocol reverse engineering analysis.

## Introduction

### Research background and significance

With the evolution and development of communication technologies, a large number of proprietary communication protocols have been designed and deployed in various network systems to meet specific performance and functional requirements. These protocols typically lack publicly available specifications. While their proprietary and confidential nature helps safeguard the security of specialized operations, it also poses a dual challenge for cyberspace security management: on one hand, security analysts find it difficult to effectively monitor and assess vulnerabilities in traffic that uses proprietary protocols; on the other hand, attackers may exploit their closed nature to launch malicious attacks, thereby evading detection and posing a serious threat to communication infrastructure security. In this context, protocol reverse engineering, as a key technology for understanding and analyzing the core logic of unknown protocols, has become an important research direction in the field of cybersecurity [1]. Among its tasks, protocol state machine inference, serving as the core step of protocol reverse engineering, aims to automatically reconstruct the session state model of a protocol from network traces. It reveals the protocol’s core control logic and helps understand its behavioral specifications, holding significant importance for enabling accurate traffic analysis [2], vulnerability discovery [3], and intrusion detection [4].

### Research status

#### Overview of protocol state machine inference.

A protocol state machine characterizes the rules governing state transitions during a protocol session, serving as the foundation for understanding protocol interaction logic and conducting security analysis. Current methods for protocol state machine inference can be primarily categorized into two types based on whether they involve active interaction with the target protocol: interactive and non-interactive. Interactive methods (e.g., algorithms based on active learning), while capable of generating precise models, rely on the prerequisite that the protocol entity can be interacted with. This limits their applicability when analyzing private, closed protocols [5]. Consequently, non-interactive methods (also known as passive inference), which infer models by directly analyzing network traffic, have become the mainstream research direction in this field [6].

#### General paradigms and core challenges of non-interactive inference.

Existing non-interactive methods generally follow a serial paradigm of “state feature extraction → state machine construction → state machine optimization.” Specifically: 1. State Feature Extraction: The goal of this step is to extract key feature vectors from protocol message sequences. Existing algorithms predominantly rely on shallow statistical features (such as common subsequences [7], frequent items [8], mutual information scores [9]) or manual rule-based field matching methods. Consequently, they struggle to effectively capture the deep semantic and long-range dependencies within protocol interactions. 2. State Machine Construction: Based on the extracted features, this step first employs clustering algorithms (e.g., nearest neighbor clustering [10], hierarchical clustering [11]) to assign state labels to session segments. It then constructs an initial state machine model, such as a prefix tree acceptor [12], based on the transition relationships between messages. 3. State Machine Optimization: To address common issues in the initial model, such as state redundancy and excessive model complexity caused by inappropriate clustering granularity, this step applies simplification operations like state merging [9] and pruning [13]. These operations eliminate redundant states and transition edges, ultimately yielding a concise and robust state machine representation.

Although this paradigm has achieved initial progress, its inherent structural flaws severely constrain further improvement in inference performance, primarily manifested in the following three aspects:

1. Feature Representation Bottleneck: Existing methods excessively rely on manually-defined shallow statistical features or rules, making it difficult for them to automatically learn highly discriminative deep semantic features from raw interaction sequences. Consequently, they fail to effectively model the crucial long-range dependencies in protocol behavior.
2. Inter-stage Representation Fragmentation Problem: In this serial pipeline, typically only deterministic, discrete intermediate results (such as hard clustering labels) are passed between stages. This leads to significant loss of rich original information, including feature distributions and uncertainties, during transmission. As a result, subsequent stages must make decisions based on incomplete information, adversely affecting the overall model performance [14].
3. Insufficient Adaptive Capability of Optimization Strategies: The execution of core simplification operations like state merging and pruning primarily depends on pre-set thresholds or empirical heuristic rules. They lack the ability to autonomously infer and adjust optimization strategies from data specific to a particular protocol. Therefore, when confronted with private protocols that have novel structures and complex behaviors, existing methods struggle to make precise adaptive adjustments. Their generalization performance and robustness are significantly constrained, leading to unstable performance.

#### Representative work and its limitations.

To address the aforementioned challenges, researchers have proposed various non-interactive state machine inference methods. Early pioneering work such as ScriptGen [15], with its process of constructing a prefix tree acceptor followed by hierarchical clustering for simplification, fully embodies the “feature extraction → construction → optimization” serial paradigm. However, it relies on shallow sequence alignment features of packets, and its simplification strategy is heavily dependent on pre-set clustering parameters. Subsequently, frameworks like Prospex [16] improved the identification of state causality by synchronously modeling bidirectional information flows, yet they still transmit hard clustering labels between stages, leading to information loss. To more finely characterize state transitions, Finite State Transducers (FSTs) have been widely applied in protocol state machine reasoning in recent years. PRETT [10] and ReFSM [8] are representative works in this area. They continuously update and refine the model by explicitly recording and matching correspondences between bidirectional information, ultimately achieving more precise state machine inference. Most current methods, however, fail to fully leverage the probabilistic nature of state transitions, thereby limiting the completeness of the description of protocol dynamic behavior. Addressing this limitation, Wang et al. proposed Veritas [17], a state machine inference method based on probabilistic analysis. This method uses the cluster centers obtained from the PAM algorithm as state messages and ultimately performs state machine construction and simplification through a custom probabilistic generalization model. The PRISMA method proposed by Krueger et al. [18] first clusters messages into binary and text types, then learns the state transition probabilities of a Markov model using maximum likelihood estimation, and finally converts it into a Moore state machine and simplifies it, thereby extending the ASAP method. Sun et al. proposed a progressive protocol reverse learning method named Spita-PL [7]. It models both message interactions and probabilities using a stochastic finite state transducer model and employs a dynamic state merging strategy to avoid state explosion, achieving efficient and high-precision inference of unknown protocol behaviors.

### Main contributions

Although the aforementioned works have improved state machine inference at various levels by introducing bidirectional modeling, correspondence relationships, or probabilistic models, they have not fundamentally overcome the three major structural deficiencies: the feature representation bottleneck, inter-stage representation fragmentation, and insufficient adaptive capability of optimization strategies. To address this, this paper proposes a protocol state machine inference algorithm based on Mamba and GCN: MambaGCN-SMI. Its main contributions are as follows:

1. It proposes a deep feature extraction method based on a dual-level state space model. By employing the Mamba model for a two-level encoding structure of packet semantics and session states, it effectively captures long-range dependencies and complex contextual patterns in protocol interactions, achieving a mapping from shallow statistical features to deep semantic features.
2. It proposes a directed graph-based method for protocol state machine construction. First, clustering algorithms are used to stably map the continuous state features extracted by Mamba to discrete state labels. Then, an initial protocol state machine in the form of a weighted directed graph is constructed, using cluster centers as nodes, transitions between states as edges, and transition frequencies as weights. This fully characterizes the discrete state space and dynamic transition patterns of the protocol.
3. It introduces Graph Neural Networks into the state machine simplification stage. By leveraging the state transition graph to drive a GCN in learning low-dimensional embeddings for state nodes, the cosine distance between embeddings directly reflects the similarity of state transition distributions, thereby enabling data-driven adaptive state merging.
4. It validates the effectiveness and superiority of the method on public datasets: Through systematic experiments on typical protocol datasets such as TCP and SMTP, the proposed algorithm is verified to have significant advantages in state machine inference accuracy, structural conciseness, and predictive utility of the state machine. This demonstrates the method’s applicability and advancement in practical scenarios.

The chapter structure of this paper is arranged as follows: Chapter 2 reviews the research background and related work in protocol state machine inference. Chapter 3 elaborates in detail on the overall architecture and key design of the proposed MambaGCN-SMI method. Chapter 4 provides a systematic analysis and discussion of the experimental datasets, evaluation metrics, and results. Chapter 5 summarizes the entire work and prospects for future research directions.

## Related work

Recent breakthroughs in deep learning within key areas such as sequence modeling and graph representation learning have provided new technological pathways to overcome the aforementioned bottlenecks. Specifically, to address the fragmentation issues inherent in the traditional serial paradigm and to construct a coherent framework capable of automatically learning deep semantic features while supporting the entire state inference pipeline with a unified representation, this study selects and integrates two core models as the foundation of the new approach: the first is the Mamba model [19], suitable for efficient feature extraction from long sequences; the second is the Graph Convolutional Network (GCN) [20], proficient in capturing and modeling structural relationships within graphs. They offer potential solutions for the feature representation bottleneck and the insufficient capability of state machine simplification in the existing paradigm, respectively. This chapter will systematically elucidate their fundamental principles, core mechanisms, and emphasize their applicability and technical advantages for the task at hand in this study.

### Mamba

Mainstream sequence modeling architectures, represented by Transformer [21], face bottlenecks in computational efficiency and memory usage when processing long sequences due to the quadratic computational complexity of their attention mechanisms. To break through this limitation, researchers have been committed to exploring new paradigms that combine powerful modeling capabilities with linear computational complexity. In this context, Mamba emerges as a breakthrough solution. By introducing a selective mechanism and hardware-aware algorithms, it achieves leading performance and efficiency in long-sequence modeling tasks. The theory of Mamba is based on the Structured State Space Sequence Model (S4) [22]. S4 models an input sequence *h*(*t*) through a hidden state *x*(*t*):


$$\begin{array}{c} \begin{array}{c} h^{\prime }(t)=Ah(t)+B(x)\\ y(t)=Ch(t) \end{array} \end{array}$$ (1)

where A is the state transition matrix, governing the propagation of information across long sequences; *B* and *C* are projection matrices. However, the time-invariant nature of the S4 model limits its context-aware capabilities. The core innovation of Mamba lies in introducing a selection mechanism based on the input sequence, which associates the parameters (B,C,Δ) with the input *x*(*t*). This enables the model to dynamically choose to re*t*ain or forget input information, endowing it with context-learning abilities comparable to those of attention mechanisms. Simultaneously, Mamba employs a parallel scan algorithm to reorganize the computation order during training, leveraging the powerful parallel computing capabilities of hardware. This results in more efficient training and inference compared to attention mechanisms.

Mamba demonstrates significant advantages in long-sequence modeling, endowing it with the potential to serve as a high-performance feature extractor for protocol state machine inference tasks. Compared to traditional recurrent neural networks or attention mechanisms whose computational efficiency is limited on long sequences, Mamba’s core mechanism, based on selective state spaces, enables it to dynamically model long-range contextual dependencies within protocol interaction sequences with linear computational complexity. Accurately capturing such dependencies is a crucial prerequisite for achieving precise discrimination of protocol states and a correct understanding of their logical evolution.

### Graph convolutional network

Traditional deep learning models (such as Convolutional Neural Networks, CNN, and Recurrent Neural Networks, RNN) excel at processing Euclidean data with regular grid structures (e.g., images) or sequential structures (e.g., text), but they struggle to effectively model non-Euclidean graph-structured data directly, such as social networks or knowledge graphs. GCNs address this by generalizing the convolution operation from regular domains to the graph domain. Their core idea is to iteratively update node representations by aggregating information from a node’s neighbors, thereby achieving representation learning for graph-structured data. GCNs have now become a fundamental framework for handling relational data. Specifically, a GCN learns layer-wise abstracted topological representations for nodes within stacked multi-layer networks by iteratively performing message passing, neighborhood aggregation, and node state updates. Through this mechanism, a GCN can generate a low-dimensional embedding vector for each node in the graph. This vector integrates the node’s own features with its local and global topological context. This makes GCN a powerful tool for analyzing and mining complex relational structures.

Recent advances have further extended GCNs by incorporating domain-specific knowledge. Yu et al. [23] proposed a physics-informed GCN that embeds dynamical system constraints—formulated as differential equations—into message passing, improving prediction fidelity in complex networks. This is particularly relevant to protocol analysis, as protocol state machines also obey formal semantic constraints (e.g., deterministic transitions, state invariants) that could be viewed as a kind of “protocol physics.” Meanwhile, Shang et al. [24] highlighted the importance of preserving topological heterogeneity, arguing that conventional smoothing-based message passing can erase critical structural diversity. Their observation that model performance varies systematically across graph density regimes resonates with our finding that feature effectiveness differs across protocols with varying state graph densities. Building on this foundation, recent extensions such as hypergraph neural networks [25] have been proposed to capture higher-order relationships beyond pairwise interactions, which may offer further potential for modeling complex protocol behaviors.

A network protocol’s state machine can be modeled as a directed graph, with states as nodes and transitions as directed edges. The initial state machine inferred directly from network traffic often contains redundant states, leading to state explosion. The core challenge in simplification lies in how to accurately measure and merge states that are topologically or behaviorally similar. GCN’s ability to learn node embeddings based on graph structure offers a data-driven solution: quantify the similarity between learned embeddings to guide merging decisions.

### MambaGCN-SMI

**Fig 1:**
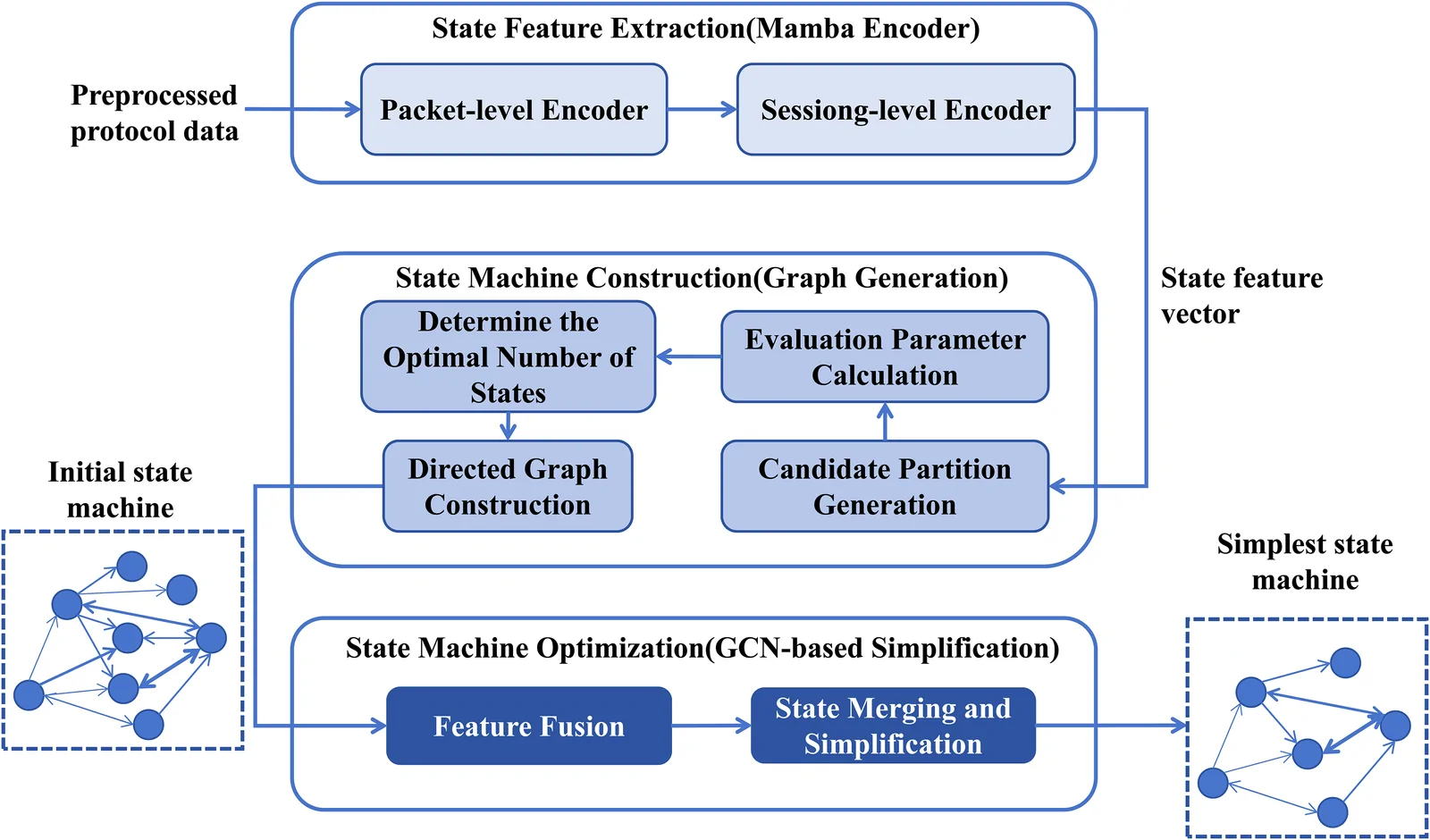
Framework diagram of MambaGCN-SMI.

The proposed MambaGCN-SMI framework adopts the serial processing paradigm of “state feature extraction → state machine construction → state machine optimization,” achieving end-to-end automated inference from raw protocol data to a concise state machine. The overall architecture is illustrated in Fig 1.

First, the feature extraction module employs a dual-level Mamba encoder to encode the raw protocol interaction sequences. Benefiting from its selective state space mechanism, this module captures long-range contextual dependencies within protocol interactions and outputs continuous session embeddings with deep semantic information. The state construction module then takes these embeddings and constructs the initial protocol state machine as a directed graph through four steps: generating candidate partitions, computing an evaluation function, determining the optimal number of clusters, and constructing the directed graph. Finally, the state machine optimization module introduces a GCN to simplify the directed graph structurally. This module aggregates neighborhood topological information of each state node through GCN operations, generating embeddings that incorporate structural context. Based on these embeddings, it calculates state similarity and adaptively merges states with similar behavioral patterns, ultimately outputting a concise and accurate final state machine.

### Dual-level encoder based on Mamba

**Fig 2:**
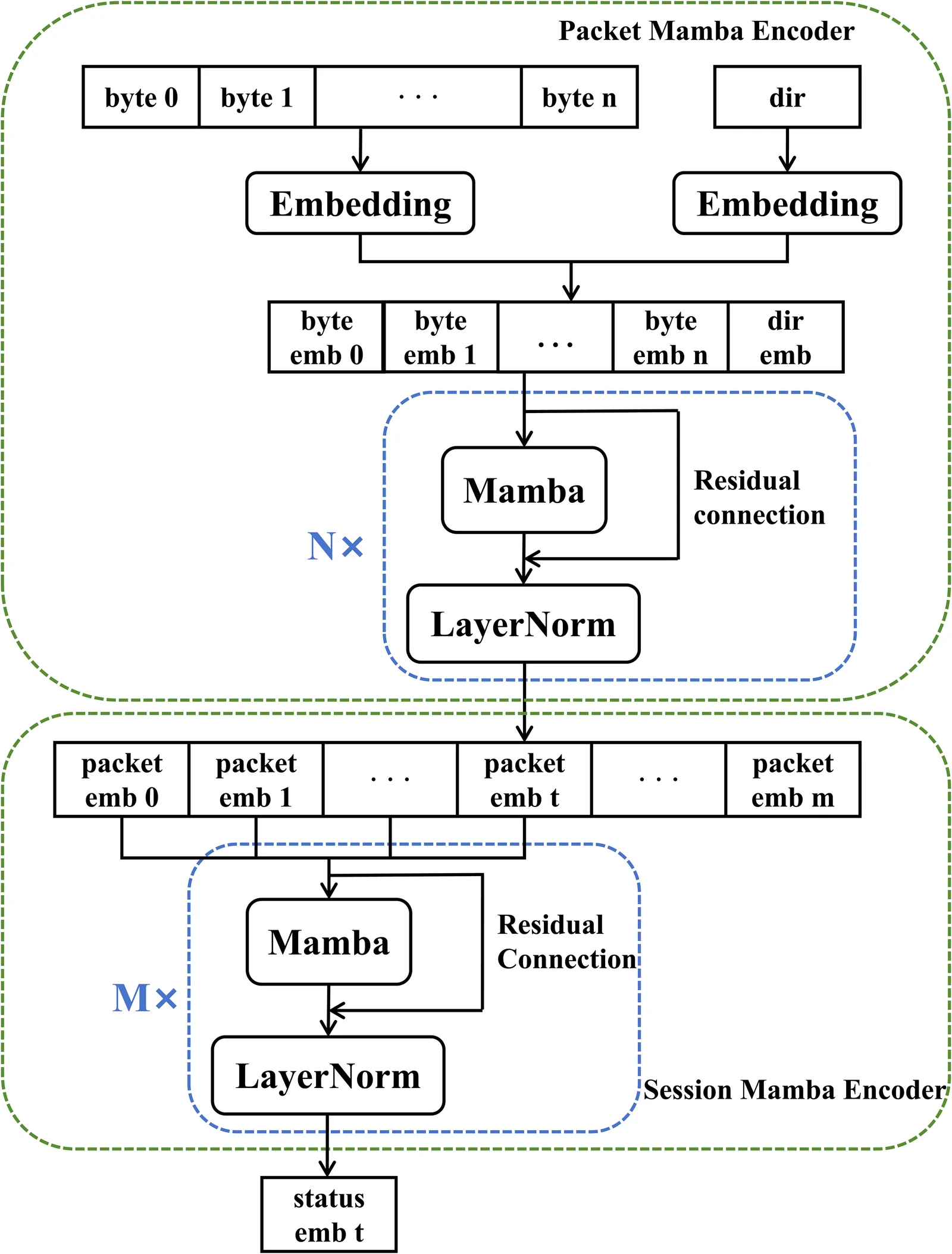
Flowchart of the two-level encoder based on Mamba.

Protocol state feature extraction derives semantic information from raw protocol interaction sequences to characterize the current session state. To this end, this section proposes a dual-level Mamba encoder. By cascading packet-level and session-level encoding, it hierarchically maps raw packet sequences to a unified state feature representation. The overall architecture is shown in Fig 2 and comprises the following two levels:

#### Message-level encoder: Message embedding.

In protocol reverse engineering, protocol messages are byte-based sequential data, where each byte (ranging from 0 to 255) is the fundamental semantic unit. The arrangement of these bytes determines the syntactic structure and high-level semantics of the message. Using raw byte values directly fails to capture semantic relationships between bytes, while one-hot encoding suffers from high dimensionality and sparsity. Therefore, this paper introduces learnable byte embeddings that map discrete bytes to low-dimensional dense vectors, compressing the representation while preserving semantic information. This aligns with recent advances in categorical-to-numerical data representation [26], which emphasize transforming discrete symbols into meaningful continuous embeddings for deep learning-based sequence modeling. Furthermore, to model the bidirectional nature of protocol interactions, directional embeddings are introduced to distinguish requests from responses.

The embedding and encoding process is as follows: Given a packet, its header is truncated or zero-padded to a fixed length L, yielding a unified byte representation sequence


$$\begin{array}{c} \begin{array}{c} byte\_emb=Embdding(BYTE)\\ dir\_emb=Embdding(dir) \end{array} \end{array}$$ (2)

where byte_emb is the embedding representation of the packet byte values, and dir_emb is the embedding representation of the packet direction. The byte embedding and direction embedding are summed to obtain the overall embedding representation x=byte_emb+dir_emb for this packet. Subsequently, this representation undergoes deep feature extraction through N layers of Mamba blocks. Each layer includes the core Mamba operation, LayerNorm for layer normalization, and residual connections to enhance training stability and representational capacity. This process can be formally expressed as:


$$\begin{array}{c} \begin{array}{c} h^{\left(l\right)}=LayerNorm(h^{\left(l-1\right)}+Mamba(h^{\left(l-1\right)})),l=1,...,N \end{array} \end{array}$$ (3)

where $h_{\;}^{\left(0\right)}=x$. Finally, the embedding vector for this packet is the output of the last layer: $packet\_emb=h_{\;}^{\left(n+1\right)}$.

This method embeds byte and directional information into a unified low-dimensional semantic space and performs deep sequence modeling on it. It provides fixed-length, semantically dense input for downstream tasks and learns contextual dependencies between protocol fields and direction-aware semantics in an end-to-end manner, enhancing representation and inference capabilities for unknown protocols.

#### Session-level encoder: Current state feature extraction.

After the packet-level encoder extracts and outputs the unified semantic vector sequence for each packet, these vectors are fed into the session-level encoder to model the temporal dependencies and state evolution of the entire session. This encoder receives an embedding vector sequence $\left[packet\_emb_{0},packet\_emb_{1},...,packet\_emb_{m}\right]$ formed by all packets within a session, where each embedding vector is a fixed-dimensional representation obtained from the packet-level encoding.

Unlike the packet-level encoder, which performs bidirectional modeling of all bytes, the session-level encoder adheres to a causal constraint: for each status t in the sequence, encoding is based solely on the preceding packet embeddings $packet\_emb_{i<t}$. This is because the protocol’s state at time t depends only on historical interactions and should not incorporate future information. This process conducts deep feature extraction through *M* layers of causal Mamba blocks. Each layer similarly includes a Mamba module, a LayerNorm layer, and residual connections. Specifically, the computation at the *l*-th layer can be expressed as:


$$\begin{array}{c} \begin{array}{c} H^{\left(l\right)}=LayerNorm(H^{\left(l-1\right)}+Mamba^{\left(l\right)}(H^{\left(l-1\right)})),l=1,...,M \end{array} \end{array}$$ (4)

where *H*^(0)^ is the embedding vector of the input packets. Unlike the common practice of only extracting the final hidden state, this model retains the intermediate state at each timestep during encoding. For the t-th packet in the input sequence, the hidden state obtained after *M* layers of causal encoding is denoted as:


$$\begin{array}{c} \begin{array}{c} status\_emb_{t}=H^{\left(M\right)}[packet\_emb_{0},packet\_emb_{1},...packet\_emb_{t} \end{array} \end{array}$$ (5)

This vector integrates all historical interaction information up to and including the current packet, forming a semantic representation of the protocol state at the current position. It can be directly used for subsequent state partitioning and state machine inference.

#### Optimization objective and loss function.

To enable the Mamba encoder to learn highly discriminative and predictive protocol state features, this work designs a joint optimization objective. This objective aims to guide the state vectors status_emb generated by the model to simultaneously satisfy two key properties: (1) Discriminability: the ability to accurately distinguish between different protocol behavior patterns; and (2) Predictability: containing sufficient information to predict subsequent interactions. To this end, a hybrid loss function *L* is introduced, combining a prediction loss $L_{pred}$ and a contrastive loss $L_{con}$.


$$\begin{array}{c} \begin{array}{c} L_{pred}=\frac{1}{B}\sum_{i=1}^{B}{\left\|status\_emb_{t}^{\left(i\right)}-packet\_emb_{t+1}^{\left(i\right)}\right\|}_{2}^{2} \end{array} \end{array}$$ (6)

$$\begin{array}{c} \begin{array}{c} L_{con}=-\frac{1}{B}\sum_{i=1}^{B}\log\frac{\exp(sim(status\_emb_{t}^{\left(i\right)},packet\_emb_{t+1}^{\left(i\right)})/\tau )}{\sum_{j=1}^{B}\exp(sim(status\_emb_{t}^{\left(i\right)},packet\_emb_{t+1}^{\left(i\right)})/\tau )} \end{array} \end{array}$$ (7)

$$\begin{array}{c} \begin{array}{c} L=L_{pred}+L_{con} \end{array} \end{array}$$ (8)

where *B* is the batch size, $sim(status\_emb_{t}^{\left(i\right)},packet\_emb_{t+1}^{\left(i\right)})$ denotes the cosine similarity, and τ is the temperature hyperparameter, set to 0.1 here, and *lambda* is the balance factor, which is used to balance the two losses and prevent one of them from dominating the training process.

The prediction loss minimizes the mean squared error (MSE) between the state vector and the embedding vector of the actual next packet, ensuring that the state vector contains sufficient information to anticipate subsequent interactions. The contrastive loss, formulated using the InfoNCE (Info Noise-Contrastive Estimation) framework [27], reduces the distance between positive sample pairs in the feature space while increasing the distance from negative samples within the same batch. Optimizing this combined loss enables the learned state vectors to predict protocol dynamics accurately while maintaining favorable inter-class separability, providing a robust foundation for subsequent unsupervised state clustering.

### Initial state mechanism construction based on directed graphs

A protocol state machine can be formally represented as a directed graph structure *G* = (*V*,*E*,*W*), where the node set *V* denotes the protocol states, the directed edge set *E* represents the transition relationships between states, and *W* is the set of edge weights, recording the frequency or probability of state transitions. To leverage the structural learning and representation capabilities of Graph Neural Networks (GNNs) [28] for data-driven adaptive state simplification, we first convert the unlabeled protocol session sequences into a structured directed graph. This section describes this transformation through the following four steps:

1. Candidate Partition Generation: First, within a predefined candidate range $\left[K_{\min},K_{\max}\right]$, candidate partitions based on state features are generated for each candidate value *K* sequentially.

We employ an agglomerative hierarchical clustering algorithm [29]. The algorithm starts with each sample as an individual cluster, continuously computes inter-cluster distances, and merges the two closest clusters until the number of remaining clusters equals the current candidate value *K*, thereby generating the corresponding state label sequence and partition scheme. To measure the semantic similarity between states more precisely, this paper selects the average linkage method as the inter-cluster distance metric and adopts cosine distance as the sample-to-sample distance metric. This focuses on the directional information of the feature vectors, reducing interference from non-semantic factors in vector norms. For two given clusters $C_{a}$ and $C_{b}$, the formula for calculating their average inter-cluster distance is as follows:


$$\begin{array}{c} \begin{array}{c} D_{\cos}(C_{a},C_{b})=\frac{1}{m\cdot n}\sum_{x_{i}\in C_{a}}\sum_{y_{j}\in C_{b}}1-\frac{x_{i}\cdot y_{j}}{\left\|x_{i}\right\|\left\|y_{j}\right\|} \end{array} \end{array}$$ (9)

2. Evaluation Metric Calculation: Traditional evaluation methods such as the elbow method [30] focus on the compactness of static feature distributions and are inadequate for characterizing the dynamic logic of protocol state transitions. Protocol interaction is a state sequence whose evolution can be described by a Markov chain. This paper employs state transition conditional entropy as an evaluation metric to assess candidate partitions from a dynamic perspective.

Specifically, for a given candidate cluster number *K* and its corresponding state partition, all protocol sessions are first transformed into state label sequences. State transition frequencies are then counted to estimate the transition probability matrix $P\in {\mathbb{R}}^{K\times K}$, where $P(s_{j}|s_{i})$ denotes the conditional probability of transitioning from state $s_{i}$ to state $s_{j}$. Concurrently, the steady-state probability $Q(s_{i})$ of a state is approximated by its occurrence frequency [26]. The state transition regularity metric *I*_reg_(*K*) is defined as the negative value of the overall state transition conditional entropy:


$$\begin{array}{c} \begin{array}{c} I_{reg}(K)=-\sum_{s_{i}\in S}Q(s_{i})\sum_{s_{j}\in S}P(s_{j}|s_{i})\log_{2}P(s_{j}|s_{i}) \end{array} \end{array}$$ (10)

A larger value of this metric indicates higher determinism and lower randomness in the state transition sequence, meaning the partition scheme better aligns with the protocol’s internal logic.

3. Determining the Optimal Number of States: The curve of the state transition regularity metric *I*_reg_(*K*) versus the cluster number *K* typically exhibits a unimodal shape. The peak point of this curve corresponds to the number of states that achieves an optimal balance between “distinguishing different behavior patterns” and “maintaining logical coherence.” Therefore, the optimal number of states $K^{*}$ can be automatically determined by identifying the maximum value of this metric:

$$\begin{array}{c} \begin{array}{c} K^{*}=\text{arg}\max_{K\in [K_{\min},K_{\max}]}(I_{reg}(K)) \end{array} \end{array}$$ (11)

This method achieves fully data-driven automatic decision-making. The system iterates through all candidate *K* values, computes and compares *I*_reg_(*K*), thereby determining $K^{*}$. This process requires no pre-set thresholds or manual intervention, ensuring the objectivity and reproducibility of the selection.

4. Directed State Transition Graph Construction: Based on the determined optimal state number $K^{*}$ and the final state labels, the initial directed graph *G* = (*V*, *E*, *W*) is constructed: (1) Node Generation and Feature Matrix Construction: Each unique state label is mapped to a node v∈V. The total number of nodes is $|V|=K^{*}$, and the corresponding cluster center feature vector for each node is taken as the node feature matrix *X*. (2) Edge Generation and Weight Statistics: The state label sequences of all sessions are scanned sequentially. For each adjacent state pair $\left(s_{t},s_{t+1}\right)$, a directed edge e∈E from node $v_{s_{t}}$ to node $v_{s_{t+1}}$ is created or updated in the graph, and its corresponding weight $w_{ij}$ is updated. The weight $w_{ij}$, as an attribute of edge $e_{ij}$, records the frequency of this state transition.

Through the above four-step process, the resulting directed graph *G* comprehensively characterizes the topological relationships and statistical patterns among protocol states, providing direct and rich structural input for the next stage of GCN-based adaptive state simplification. Through the above four steps, the resulting directed graph *G* characterizes the topological relationships and statistical patterns among protocol states, providing structural input for the subsequent GCN-based state simplification.

### Protocol state machine simplification based on graph convolutional network

The initial state machine constructed from the directed graph often contains excessive state nodes due to redundant clustering granularity, compromising conciseness and generalization. Traditional simplification methods rely on predefined thresholds or heuristic rules and lack the capacity to learn adaptively from data. To address this, we propose a GCN-based data-driven simplification method. It learns low-dimensional semantic embeddings of state nodes within the transition topology and automatically merges states with similar behavioral patterns based on embedding similarity.

#### Feature fusion.

The model input consists of the directed graph *G* = (*V*, *E*, *W*) constructed from the initial state machine and its node feature matrix *X*, where the node features are deep semantic vectors corresponding to the states, and the directed edges and their weights *W* encode the transition frequencies between states. The model utilizes a multi-layer GCN, employing a message-passing mechanism to enable the representation of each state node to aggregate transition information from its multi-hop neighbors, ultimately outputting a low-dimensional semantic embedding matrix *H*^(*L*)^.

For an L-layer GCN, its forward propagation process is generically defined as:


$$\begin{array}{c} \begin{array}{c} H^{\left(l\right)}=ReLU(\tilde{A}V^{\left(l-1\right)}W^{\left(l-1\right)}+b^{\left(l-1\right)}) \end{array} \end{array}$$ (12)

where $H^{\left(0\right)}=\tilde{X}\in {\mathbb{R}}^{K^{*}\times d}$ is the normalized node feature matrix, $\tilde{A}$ is the symmetrically normalized adjacency matrix constructed based on the weight matrix *W*, *W*^(*l*)^ and *b*^(*l*)^ are the trainable parameter matrix and bias vector for the *l*-th layer, respectively, and ReLU is the activation function. The final output $H=H^{\left(L\right)}\in {\mathbb{R}}^{K^{*}\times d}$ is the embedding matrix for all state nodes.

where $H^{\left(0\right)}=\tilde{X}\in {\mathbb{R}}^{K^{*}\times d}$ is the normalized node feature matrix, $\tilde{A}$ is the symmetrically normalized adjacency matrix constructed based on the weight matrix *W*, *W*^(*l*)^ and *b*^(*l*)^ are the trainable parameter matrix and bias vector for the *l*-th layer, respectively, and ReLU is the activation function. The final output $H=H^{\left(L\right)}\in {\mathbb{R}}^{K^{*}\times d}$ is the embedding matrix for all state nodes.


$$\begin{array}{c} \begin{array}{c} {\overset{⏜}{y}}_{i}=softmax(H_{i}\cdot W_{p}+b_{p}) \end{array} \end{array}$$ (13)

where $W_{p}\in {\mathbb{R}}^{d\times \left|V\right|}$ and $b_{p}\in {\mathbb{R}}^{\left|V\right|}$ are trainable parameters. The loss function employs cross-entropy loss, encouraging the model’s predicted transition distribution to approximate the true distribution:


$$\begin{array}{c} \begin{array}{c} L_{GCN}=-\frac{1}{\left|V\right|}\sum_{i=1}^{\left|V\right|}\sum_{j=1}^{\left|V\right|}y_{ij}\log\;{\overset{⏜}{y}}_{ij} \end{array} \end{array}$$ (14)

By minimizing ${ℒ}_{\text{trans}}$, the node embeddings *H* learned by the GCN can effectively encode the dynamic patterns of state transitions, thereby providing a highly discriminative representational foundation for subsequent state merging.

#### State merging and simplification.

After obtaining the low-dimensional semantic embeddings $H\in {\mathbb{R}}^{K^{*}\times d}$ for all state nodes, clustering is applied to merge nodes and achieve state machine simplification. This process reuses the pipeline described in Section 3.2, with the core distinction being that the input for clustering is the learned node embedding vectors, rather than the original session features.

The specific steps are as follows: 1. Node Merging: Based on the cosine distance matrix of the embedding vectors, agglomerative hierarchical clustering (average linkage) is employed to merge the state nodes. 2. Determining the Degree of Merging: The state transition regularity metric *I*_reg_ is consistently used to evaluate different merging granularities. The clustering number $K_{sim}^{*}$ that maximizes *I*_reg_ is automatically selected as the final merging result, ensuring that the simplified state machine maintains optimal determinism in its dynamic behavior. 3. State Machine Reconstruction and Pruning: The state sequences are remapped and rewritten according to the merging results, and the simplified state transition graph $G_{sim}=(V_{sim},E_{sim},W_{sim})$ is obtained through statistical analysis. To further enhance the model’s conciseness and robustness, edges in $G_{sim}$ with transition frequencies below the threshold $\theta_{freq}=0.0001$ are pruned. Nodes that lose all edges as a result of pruning are also removed to eliminate noise interference.

The nodes and edges of the final simplified state machine transition graph constitute the protocol state machine structure graph $G_{pred}$ inferred by this method. This pipeline achieves adaptive simplification of the protocol state machine, significantly reducing model complexity while preserving its core logic.

## Experiments and results analysis

### Experimental datasets

To evaluate the performance and generalization of MambaGCN-SMI, we construct a dataset comprising both standard public protocols and proprietary industrial control protocols. Its composition is detailed in Table 1. Due to comprehensive session coverage, all theoretically possible state transitions are observed for each protocol, resulting in fully connected state graphs with transition sparsity equal to 1.0. The inference task thus focuses on modeling transition probabilities rather than predicting transition existence.

**Table 1:** Experimental dataset.

Protocol	Source	Packets	Sessions	Size	States	Transitions	Rare States
TCP	WRCCDC 2024	348236	13130	567.2MB	11	19	5 (45.5%)
HTTP	WRCCDC 2024	32589	8183	78.5MB	15	19	7 (46.7%)
SMTP	WRCCDC 2024	84169	3642	14.7MB	4	13	1 (25%)
Modbus	CSET 2016	64333	3072	24.6MB	14	24	4 (28.6%)
DNP3	Github	12227	1976	8.6MB	18	34	5 (27.8%)

*Note:* Rare states are those with frequency < 5% of total samples.

Public Internet Protocols: TCP, HTTP, and SMTP are selected as representative protocols. These protocols have clear RFC specifications and well-established state machine logic, serving as benchmarks for verifying algorithm performance and facilitating horizontal comparison. The data comes from the WRCCDC 2024 public dataset [31], generated in a real-world capture-the-flag (CTF) environment with rich and dynamic network interactions, offering high authenticity and representativeness.

Proprietary Industrial Control Protocols: Modbus/TCP and DNP3 are included as test subjects. Both are widely used in critical infrastructure such as power and manufacturing. Their protocol logic is often tightly coupled with physical processes, and publicly available documentation is limited. This category tests the method’s robustness in real-world, documentation-scarce scenarios. The Modbus/TCP [32] and DNP3 [33] data come from public datasets, containing various interaction sessions from normal operations to anomaly injections, which support evaluation of the method’s inference capability on proprietary protocols.

To evaluate MambaGCN-SMI systematically, we process the original PCAP files of the above protocols using Wireshark [34]. We extract the protocol headers and their corresponding labels, constructing a multi-layer test dataset covering both public standard protocols and proprietary industrial protocols. For model training and evaluation, the data for each protocol is randomly split into training and validation sets at an 85:15 ratio. The training set is used to infer the state machine, and the validation set tests its predictive capability on unseen packets.

### Evaluation indicators

To evaluate MambaGCN-SMI, we design metrics from two aspects: state machine structure recovery accuracy and dynamic behavior prediction capability. The former measures topological recovery precision by comparing the inferred state machine against the ground truth in terms of directed graph structure. The latter treats the state machine as a predictive model, testing whether it has learned the protocol’s dynamic logic and can predict future state transitions, reflecting its real-world applicability. These two sets of metrics complement each other, forming a complete evaluation system for inference accuracy, robustness, and practicality.

#### State machine structure similarity metrics.

This category of metrics aims to quantify the differences between the inferred directed graph $G_{\text{pred}}=(V_{\text{pred}},E_{\text{pred}})$ and the ground truth directed graph $G_{\text{true}}=(V_{\text{true}},E_{\text{true}})$ at both the node and edge levels. Prior to evaluation, it is necessary to establish a semantic alignment relationship between the two types of state nodes: for each inferred state node, the most frequently occurring true protocol state label among all session samples contained within that node is assigned as its representative true label, thereby constructing a semantic mapping from inferred states to true states.

1. Node (State) Level
  - Precision: $P_{\text{node}}=\frac{\left|V_{\text{pred}}\cap V_{\text{true}}\right|}{\left|V_{\text{pred}}\right|}$, which measures the proportion of correctly matched states among the inferred states.
  - Recall: $R_{\text{node}}=\frac{\left|V_{\text{pred}}\cap V_{\text{true}}\right|}{\left|V_{\text{true}}\right|}$, which measures the proportion of true states that are correctly inferred.
2. Edge (Transition) Level
  - Precision: $P_{\text{edge}}=\frac{\left|E_{\text{pred}}\cap E_{\text{true}}\right|}{\left|E_{\text{pred}}\right|}$, which measures the proportion of correctly matched transitions among the inferred transitions.
  - Recall: $R_{\text{edge}}=\frac{\left|E_{\text{pred}}\cap E_{\text{true}}\right|}{\left|E_{\text{true}}\right|}$, which measures the proportion of true transitions that are correctly inferred.

For both nodes and edges, the composite score employs the F1-score [35], which is the harmonic mean of their respective precision and recall values:


$$\begin{array}{c} \begin{array}{c} F_{1}=\frac{2\times P\times R}{P+R} \end{array} \end{array}$$ (15)

substituting the corresponding precision and recall values into the above formula yields the node F1-score (*F*1_node_) and the edge F1-score (*F*1_edge_).

Protocol reverse engineering typically targets unknown or proprietary protocols in a blind, unsupervised setting, where ground-truth semantic labels (e.g., “LISTEN” or “ESTABLISHED”) are unavailable by definition. In such scenarios, traditional interpretability in the form of human-readable state names is inherently unattainable. The node-level metrics defined above serve as quantitative interpretability proxies. A high node F1-score indicates that the inferred states consistently correspond to distinct and stable behavior patterns, even without meaningful names. This provides structural interpretability: analysts can understand protocol behavior by observing transition patterns between these consistent states, even without explicit semantic labels.

#### Packet prediction capability.

Since the inferred state machine is a probabilistic model, its core value lies in predicting the probability distribution of the next state, rather than making a single deterministic decision. To evaluate its capability in capturing the uncertainty inherent in protocol dynamics, we design metrics at two levels:

1. Prediction Accuracy (Accuracy): This metric evaluates the model’s performance when forced to make a single decision, i.e., selecting the most probable next state from its predicted distribution and comparing it with the actual state. It measures the model’s deterministic prediction precision. Its calculation follows traditional accuracy, but the decision comes from the maximum value of the probability distribution.

$$\begin{array}{c} \begin{array}{c} Accuracy=\frac{1}{N}\sum_{i=1}^{N}\left||(\text{arg}\underset{j}{\max}P_{pred}^{\left(i\right)}(j)=s_{true}^{\left(i\right)}\right) \end{array} \end{array}$$ (16)

where *N* is the number of test samples, 𝕀(·) is the indicator function (equal to 1 if the condition is satisfied, 0 otherwise), $P_{\text{pred}}^{\left(i\right)}(j)$ is the probability predicted by the model for state *j* of the *i*-th sample, and $s_{\text{true}}^{\left(i\right)}$ is the true state of the next packet.

2. Behavior Coverage (Coverage): This metric assesses the generalization capability of the inferred state machine with respect to the true protocol behavior. Specifically, if the predicted probability of the true next state is not lower than a threshold (empirically set to 0.2), the prediction is considered to have covered the actual behavior. This metric reflects the completeness of the model’s probability distribution: higher coverage indicates a more comprehensive understanding of the protocol’s potential state transitions, and the output distribution captures the uncertainty in real interactions more accurately.

$$\begin{array}{c} \begin{array}{c} Coverage=\frac{1}{N}\sum_{i=1}^{N}\left||(P_{pred}^{\left(i\right)}(s_{true}^{\left(i\right)})>0.2\right) \end{array} \end{array}$$ (17)

#### Link prediction metrics.

The edge F1-score measures the accuracy of predicted state transitions at a discrete threshold but does not capture ranking performance or provide a threshold-free evaluation. To complement it—particularly given the sparse nature of protocol state graphs—we introduce three additional metrics commonly used in link prediction tasks:

1. AUC-ROC [36]: This metric evaluates the model’s ability to distinguish between true and false transitions across all probability thresholds. The ROC curve plots the True Positive Rate (TPR) against the False Positive Rate (FPR) at various threshold settings, where:

$$\begin{array}{c} \begin{array}{c} \text{TPR}=\frac{\text{TP}}{\text{TP}+\text{FN}},\;\text{FPR}=\frac{\text{FP}}{\text{FP}+\text{TN}} \end{array} \end{array}$$ (18)

AUC-ROC represents the probability that a randomly chosen true transition receives a higher predicted probability than a randomly chosen false transition. It is computed as:


$$\begin{array}{c} \begin{array}{c} \text{AUC-ROC}=\int_{0}^{1}\text{TPR}({\text{FPR}}^{-1}(x))\;dx=P({\hat{y}}_{\text{true}}>{\hat{y}}_{\text{false}}) \end{array} \end{array}$$ (19)

A value of 1.0 indicates perfect separation, while 0.5 indicates random guessing.

2. AUC-PR [36]: Unlike ROC, which considers both true positives and false positives, the Precision-Recall curve focuses on the positive class (actual transitions). The PR curve plots Precision against Recall at various threshold settings, where:

$$\begin{array}{c} \begin{array}{c} \text{Precision}=\frac{\text{TP}}{\text{TP}+\text{FP}},\;\text{Recall}=\frac{\text{TP}}{\text{TP}+\text{FN}} \end{array} \end{array}$$ (20)

AUC-PR summarizes the trade-off between precision and recall across different thresholds:


$$\begin{array}{c} \begin{array}{c} \text{AUC-PR}=\int_{0}^{1}\text{Precision}({\text{Recall}}^{-1}(x))\;dx \end{array} \end{array}$$ (21)

This metric is particularly informative for sparse graphs like protocol state machines, where true transitions are rare compared to all possible transitions. AUC-PR is more sensitive than AUC-ROC in imbalanced settings, as it focuses on performance on the positive class.

3. Recall@K [37]: This metric evaluates the model’s practical utility in ranking plausible next states. For each state, we rank all possible next states by their predicted probabilities and check whether the true next state appears in the top-K predictions. Recall@K is computed as:

$$\begin{array}{c} \begin{array}{c} \text{Recall@K}=\frac{1}{N}\sum_{i=1}^{N}𝕀({\text{true state}}_{i}\in {\text{top-K predictions}}_{i}) \end{array} \end{array}$$ (22)

where *N* is the number of test samples and 𝕀(·) is the indicator function. This metric is especially relevant for real-world applications where analysts may consider multiple plausible next states.

Together with the edge F1-score, these metrics provide a multi-faceted evaluation of our method’s link prediction capability: edge F1 measures threshold-based accuracy, AUC-ROC and AUC-PR assess ranking and discrimination ability, and Recall@K evaluates practical utility in top-K recommendations.

### Experimental environment

All experiments were conducted under a unified hardware and software environment: the operating system was Windows 11, equipped with an NVIDIA RTX A6000 GPU. The model implementation was based on the PyTorch 2.1.1 + CUDA 11.8 framework, while traditional clustering and evaluation components utilized Scikit-learn 1.2.2. During training, the Adam optimizer was employed with an initial learning rate set to $5\times 10^{-4}$ for a total of 200 epochs. To ensure the reproducibility and statistical stability of the experiments, all experiments were conducted with a fixed random seed and independently repeated 5 times to mitigate the effects of random fluctuations.

### Experimental environment

All experiments were conducted under a unified hardware and software environment: the operating system was Windows 11, equipped with an NVIDIA RTX A6000 GPU. The model implementation was based on the PyTorch 2.1.1 + CUDA 11.8 framework, while traditional clustering and evaluation components utilized Scikit-learn 1.2.2. During training, the Adam optimizer was employed with an initial learning rate of $5\times 10^{-4}$ for a total of 200 epochs. To ensure reproducibility and statistical stability, all experiments used a fixed random seed and were independently repeated 5 times to mitigate the effects of random fluctuations.

### Model parameter selection

**Fig 3:**
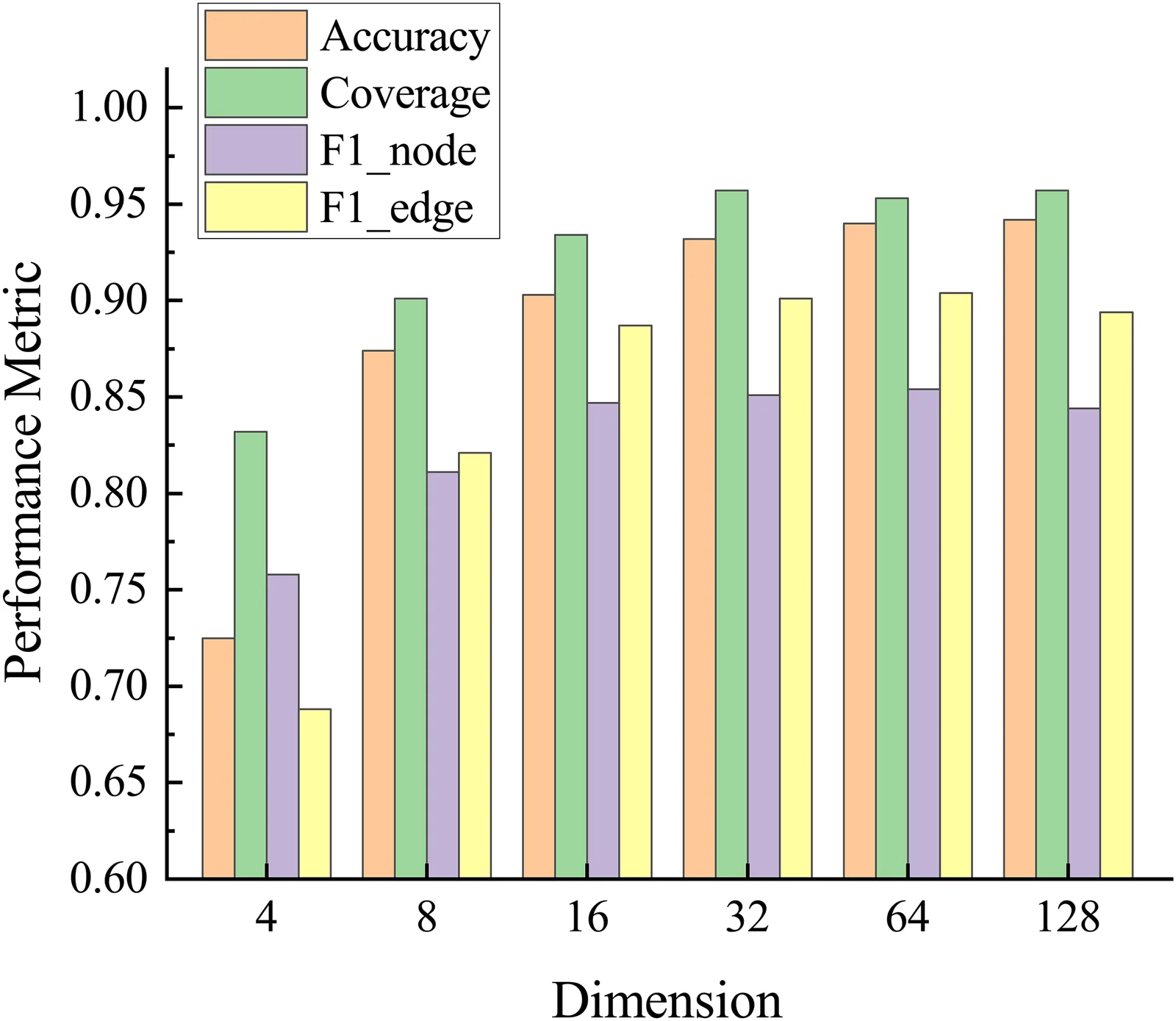
The impact of feature embedding dimension on model performance.

**Fig 4:**
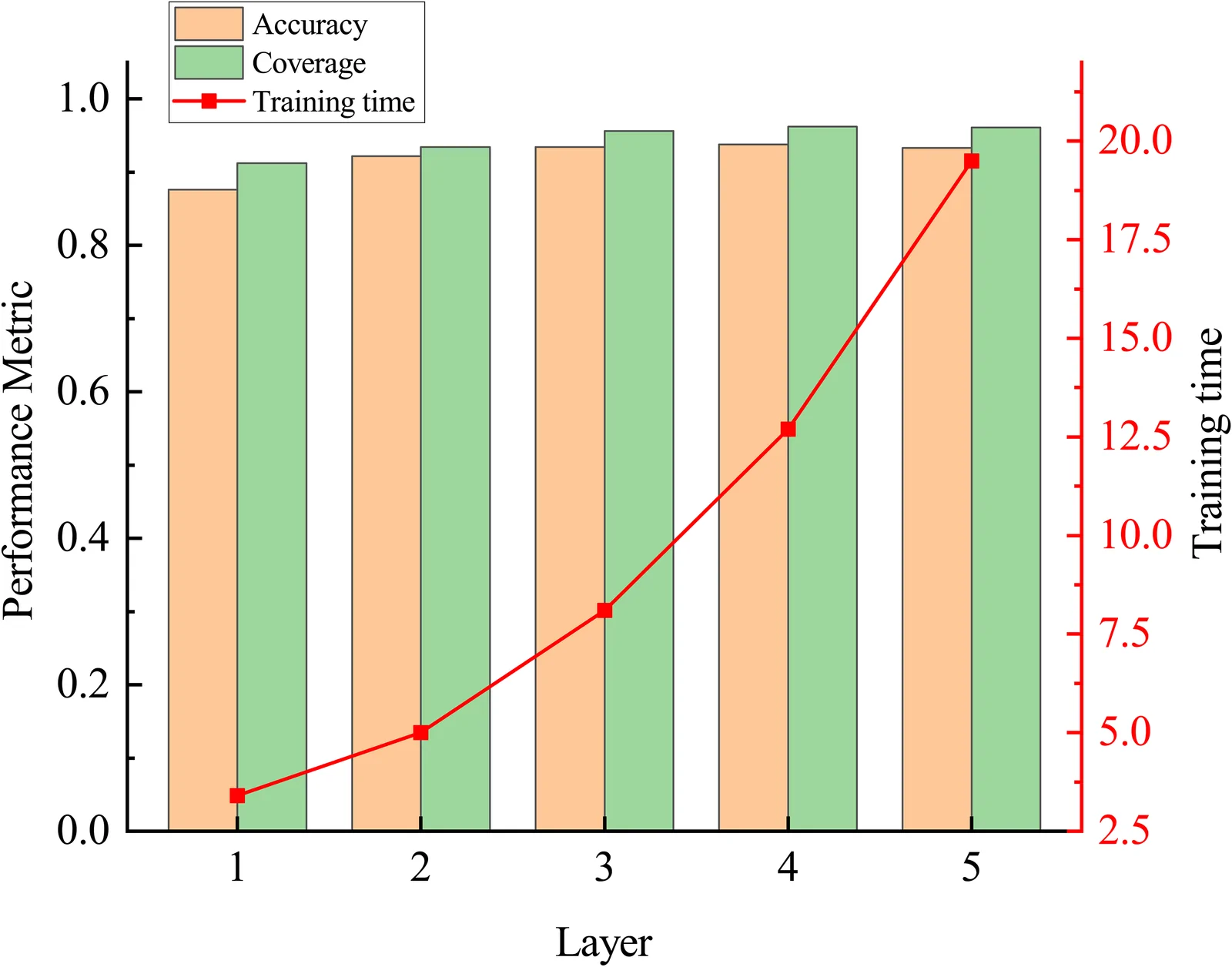
The influence of GCN layer number on performance.

To ensure the objectivity and reproducibility of the MambaGCN-SMI framework configuration and to establish a fair benchmark for subsequent comparative experiments, this section systematically tunes key hyperparameters, including the following:

1. Feature Embedding Dimension ($d_{type\_emb}$): This parameter controls the precision of state node semantic representation. Insufficient dimensionality limits semantic separability between states, reducing feature discriminability; excessive dimensionality introduces representational redundancy and noise sensitivity, increases model complexity, and may trigger overfitting. To determine the optimal dimension, we compare model performance when the embedding dimension is set to 4, 8, 16, 32, 64, and 128, focusing on state partitioning accuracy and generalization. The experimental results, shown in Fig 3, indicate that at dimension 32, the model maintains high discrimination accuracy with relatively low computational and storage costs, and no significant overfitting is observed. We therefore set the feature embedding dimension to 32. This configuration effectively captures semantic differences between states and provides a discriminative feature representation for subsequent state machine construction and simplification.
2. Mamba Encoder Architecture: The architectural parameters of the Mamba encoder—mainly the number of layers and the output feature dimension—determine the model’s feature extraction capability. The number of layers controls the depth of modeling for protocol interaction sequences, affecting the capture of long-range dependencies; the output dimension determines the capacity of the state semantic representation. To reduce model complexity and ensure consistency across feature hierarchies, the packet-level and session-level encoders share the same layer count and output dimension. This reduces the number of hyperparameters and keeps the semantic abstraction of the two levels aligned. To determine the optimal structure, we conduct a combinatorial experiment on output dimensions (16, 32, 64) and layer counts (2, 3, 4), as shown in Table 2. The results indicate that with an output dimension of 32 and 3 layers, the model achieves the best overall performance in accuracy (94.32%), coverage (96.65%), and F1-score (92.19%). However, since the subsequent state clustering and state number evaluation stages require multiple scans, a lower dimension reduces computational overhead and improves overall efficiency. Increasing the dimension to 64 yields limited performance gains while drastically increasing computational costs; performance stabilizes after 3 layers, and adding more layers tends to cause overfitting. Therefore, balancing performance and efficiency, we adopt an output dimension of 16 with 3 layers. This maintains sufficient semantic discriminability while enhancing the practicality of the inference pipeline.
3. GCN Layer Number: The number of GCN layers controls the scope of information propagation and aggregation for state nodes within the graph structure. Too few layers restrict the model’s ability to capture multi-hop state dependencies, leading to underutilization of local information; too many layers may cause over-smoothing, where features of different nodes become homogenized, weakening their semantic distinctiveness. Therefore, to determine the optimal number of GCN layers, this paper systematically tests the impact of the GCN layer number on the state machine simplification effect, with the results shown in Fig 4.

**Table 2:** The influence of Mamba encoder structure on model performance.

Output dimension	Layers	Accuracy (%)	Coverage (%)	$F_{1}^{node}$ (%)	$F_{1}^{edge}$	Training time (s)	Parameter quantity
16	2	89.42	92.10	87.55	0.812	25.42	43,680
16	3	93.91	96.18	91.02	0.857	37.21	61,376
16	4	94.30	95.81	89.61	0.855	52.16	79,072
32	2	91.05	93.45	89.38	0.823	210.60	148,800
32	3	94.32	96.65	92.19	0.859	315.89	214,912
32	4	94.40	96.64	91.83	0.854	421.23	281,024
64	2	92.88	95.28	90.51	0.835	1,685.43	543,360
64	3	94.27	96.66	91.20	0.846	2,527.74	798,464
64	4	94.79	95.92	90.77	0.851	3,370.17	1,053,568

1. Other Implementation Details: Raw PCAP files are processed using Wireshark to extract protocol headers, with each message represented as a byte sequence of maximum length *L* = 128 bytes (truncated or padded as needed). This length is selected because it covers the majority of protocol messages in our datasets-analysis shows that over 95% of messages are shorter than 512 bytes. Longer messages are rare and typically contain repetitive payload data rather than state-relevant information, so truncation does not significantly impact state inference performance. The overall loss function combines cross-entropy loss for state prediction and graph-structured loss from the GCN module, with a balancing factor λ=0.5. This value is chosen to give equal weight to both losses, as preliminary experiments showed that the model is robust to variations in the range [0.3, 0.7], with performance fluctuations below 1%. The equal weighting also simplifies interpretation and avoids introducing additional tuning complexity. For clustering-based state construction, the candidate cluster number range is set to [10–40] based on prior knowledge and preliminary experiments. This range is chosen because it sufficiently covers the typical state space of most protocols-common protocols such as TCP, HTTP, SMTP, Modbus, and DNP3 all have state counts within this interval. Moreover, the optimal number of states $K^{*}$ is automatically selected by maximizing the state transition regularity metric $I_{reg}(K)$ within this range, making the exact bounds non-sensitive: as long as the range encompasses the true state count, the automatic selection mechanism ensures a robust result. For state machine simplification, the edge pruning threshold $\theta_{freq}=0.0001$ is empirically set to remove noise transitions while preserving core behavioral patterns. This threshold is chosen based on the observation that valid state transitions consistently occur with frequencies above this value in our datasets, while spurious transitions (e.g., due to packet loss or retransmission) typically fall below it. Importantly, this parameter is not sensitive-its sole purpose is to prevent state explosion by eliminating negligible noise; as long as it is set sufficiently low, it does not affect the core structure of the inferred state machine.

### Overall performance comparison

In this section, performance testing is conducted based on the optimal parameter configuration of MambaGCN-SMI, specifically: feature embedding dimension 32; Mamba encoder output dimension 16 with 3 layers; GCN with 3 layers. Through comprehensive evaluation on the multi-protocol dataset and comparison with several advanced algorithms, the effectiveness and superiority of the proposed method for the protocol state machine inference task are systematically validated.

#### Performance of MambaGCN-SMI under optimal configuration.

**Fig 5:**
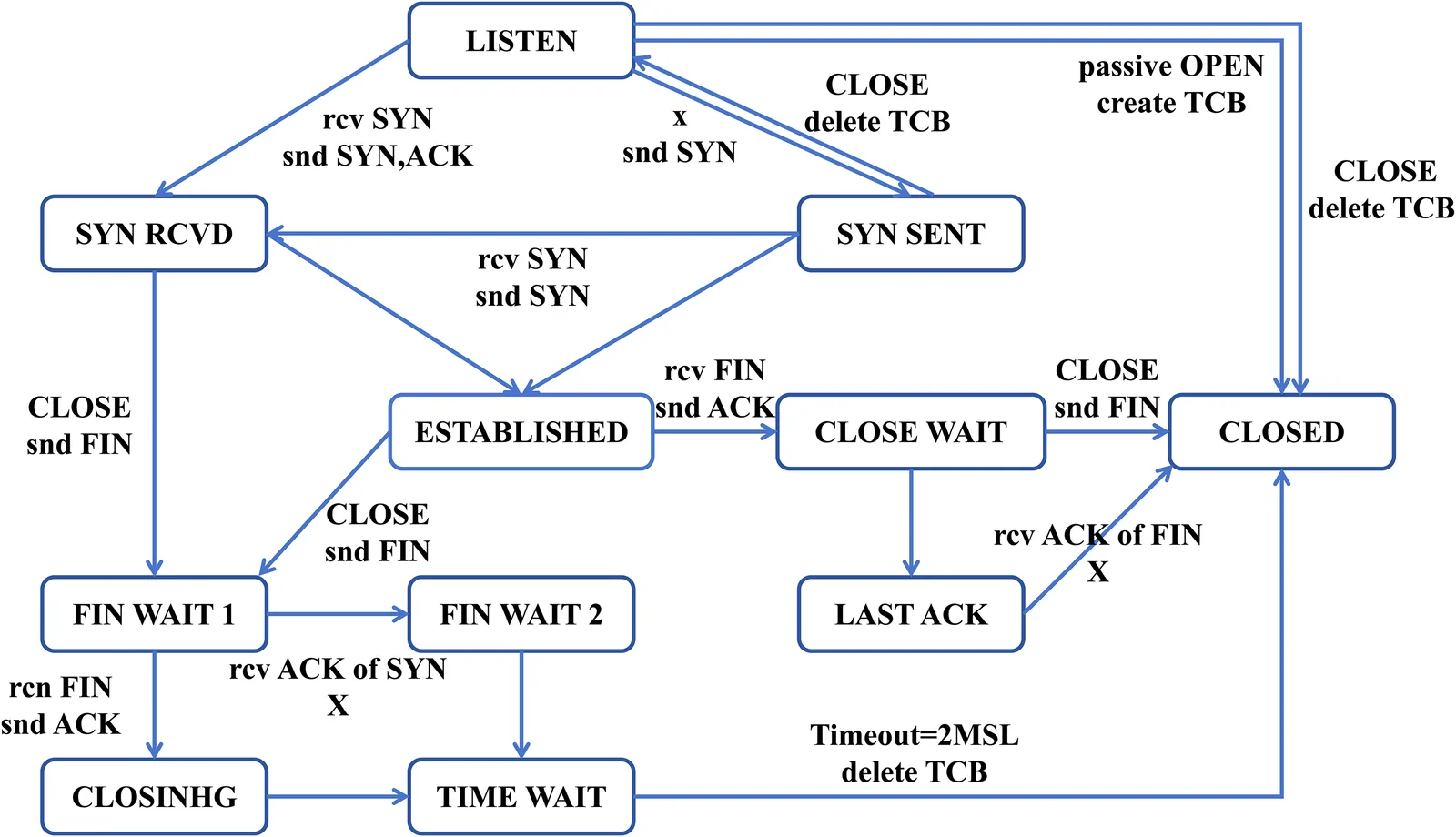
Real state diagram of TCP protocol.

**Fig 6:**
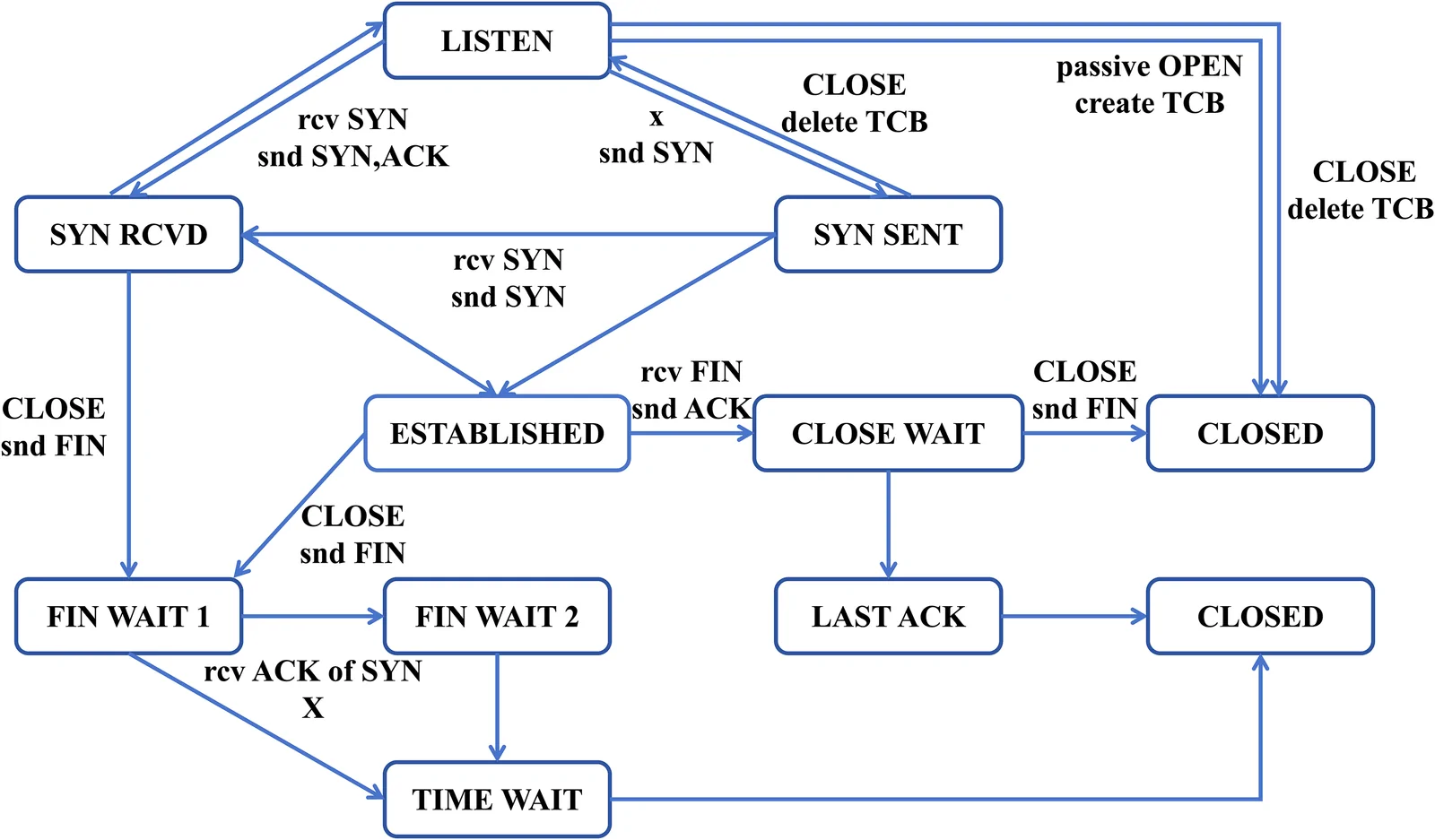
Inferred state diagram of TCP protocol.

**Fig 7:**
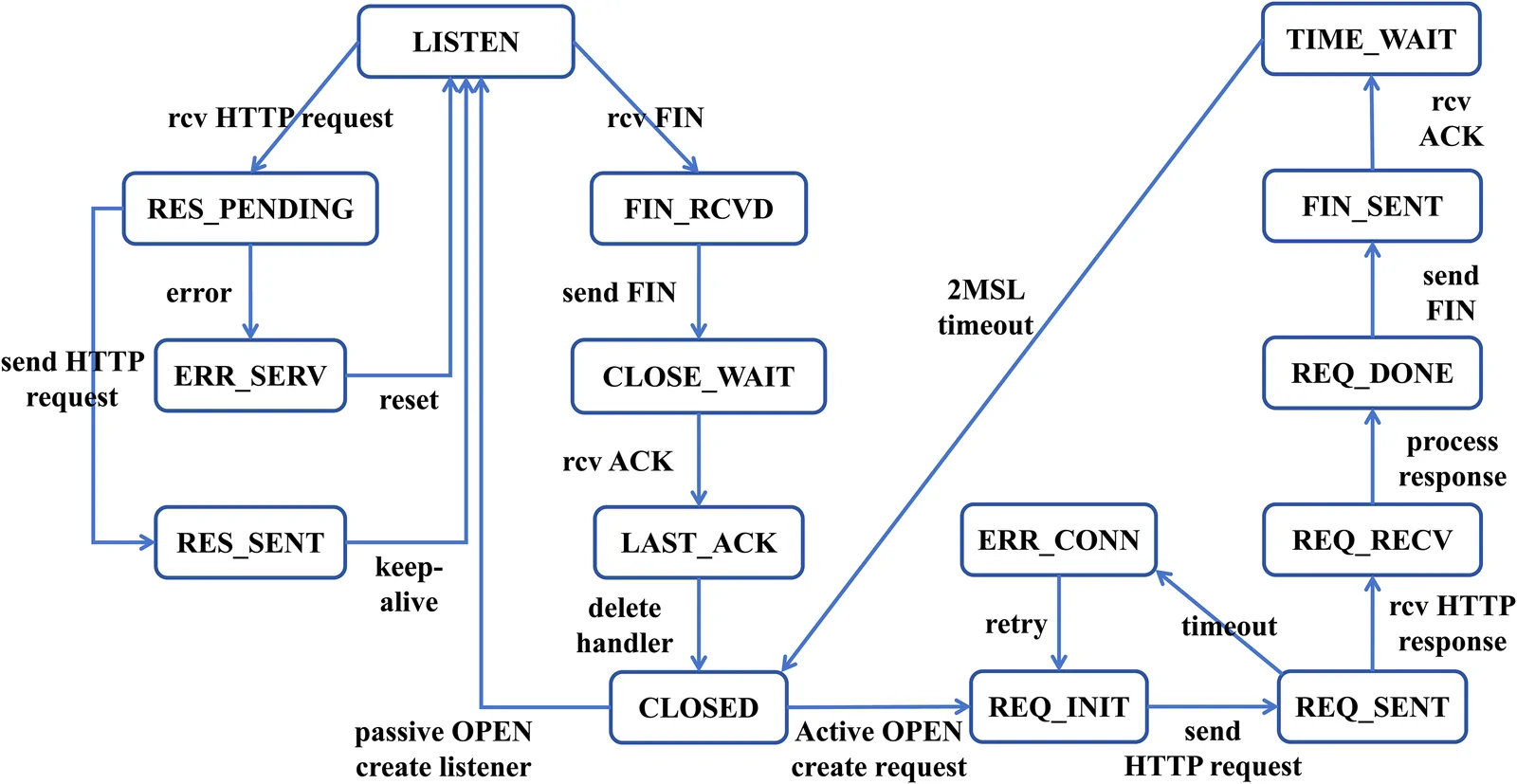
Real state diagram of HTTP protocol.

**Fig 8:**
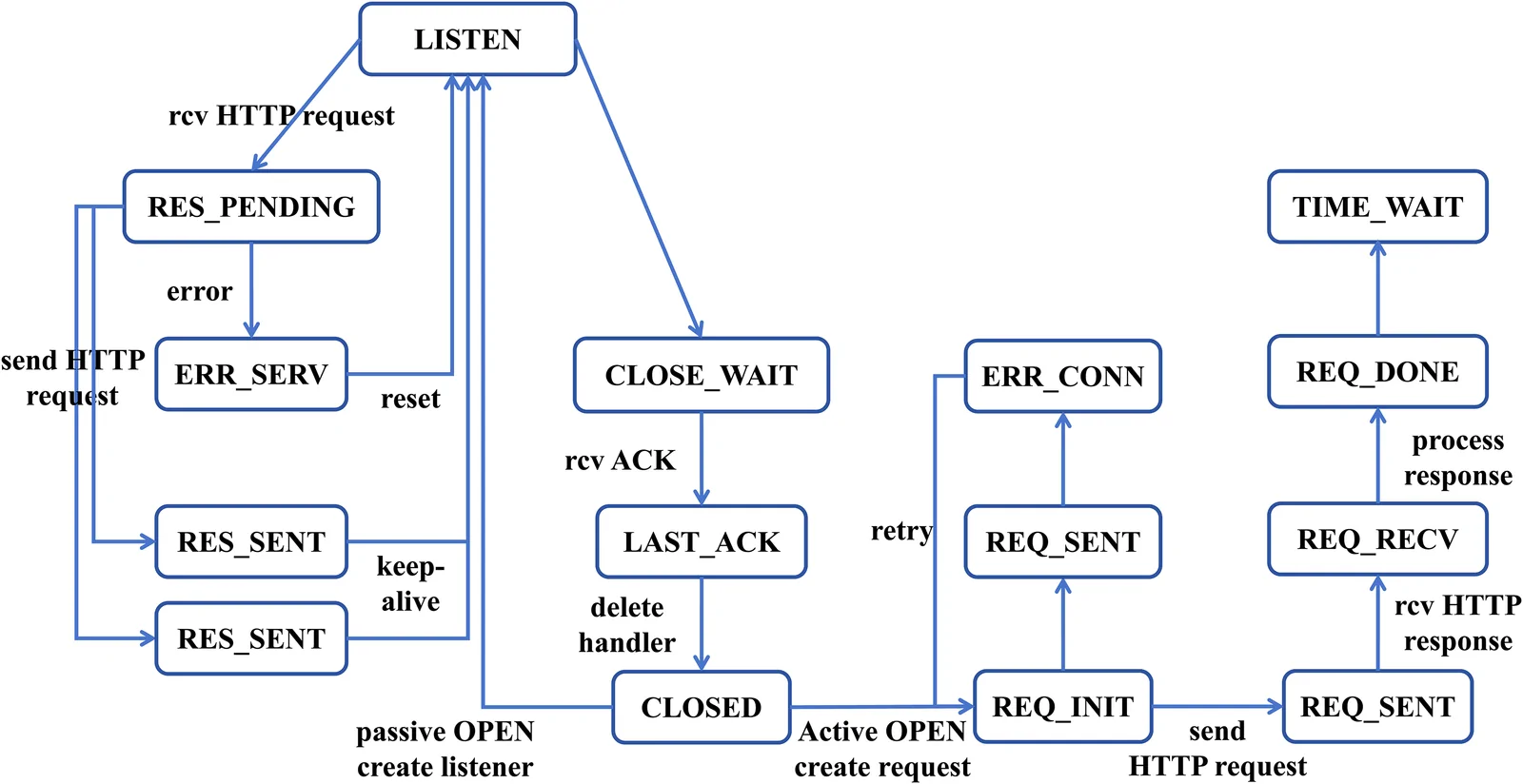
Inferred state diagram of HTTP protocol.

To intuitively demonstrate the effectiveness of MambaGCN-SMI in state machine inference, this section first uses the TCP and HTTP protocols as examples to visually compare the inferred state machines with the ground truth state machines, as shown in Figs 5–8. In the figures, nodes represent protocol states, and directed edges represent state transitions. It can be observed that for the TCP protocol, the *F*1_node_ and *F*1_edge_ of the protocol state graph inferred by MambaGCN-SMI reach 0.947 and 0.892, respectively. For the HTTP protocol, the *F*1_node_ and *F*1_edge_ of the inferred state graph reach 0.886 and 0.875, respectively. These results indicate that the proposed method can not only accurately identify core states within the protocol but also effectively recover the transition logic between states.

**Fig 9:**
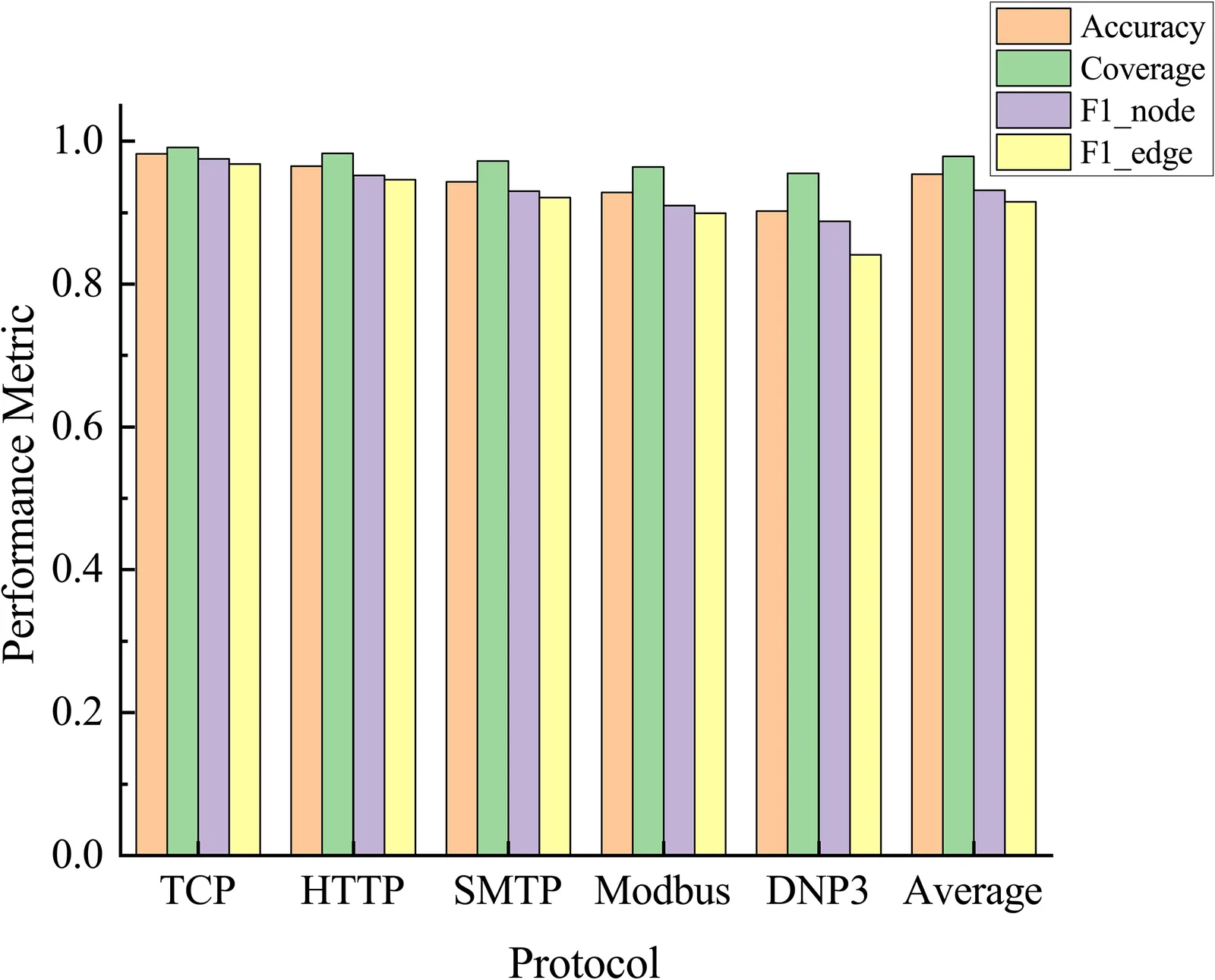
Comprehensive performance of MambaGCN-SMI.

The comprehensive performance of MambaGCN-SMI across various protocols is shown in Fig 9. Specifically: In terms of prediction performance, the model performs most prominently on the TCP and Modbus protocols, achieving Accuracy scores of 0.982 and 0.968, respectively, and Coverage scores both exceeding 0.98. This indicates that the model can effectively capture the mainstream behavioral patterns of the protocols and maintain good coverage of potential state transitions. Regarding structure recovery, on TCP and HTTP, the *F*1_node_ and *F*1_edge_ values both exceed 0.89, demonstrating that the inferred state machines are highly consistent with the ground truth state machines at the topological level. Even for the structurally complex and poorly documented industrial protocol DNP3, *F*1_node_ and *F*1_edge_ still reach 0.918 and 0.841, respectively, reflecting the method’s strong robustness in structural inference for private protocols. Overall, the algorithm maintains a high level of average comprehensive performance across the five different protocols (Accuracy: 0.957, Coverage: 0.975, *F*1_node_: 0.939, *F*1_edge_: 0.915), further validating that MambaGCN-SMI possesses good generalization capability and consistency across different protocol types and scenarios.

To provide a more comprehensive evaluation, we report the link prediction performance of MambaGCN-SMI across all five protocols in Table 3, including AUC-ROC, AUC-PR, and Recall@K (K = 1, 3, 5). The model achieves high AUC-ROC scores, with an average of 0.971, indicating its strong capability to distinguish true transitions from false ones. The average AUC-PR of 0.921 further demonstrates robust performance on the positive class, which is especially meaningful given the sparsity inherent in protocol state graphs. In terms of practical utility, the Recall@K metrics reveal that the true next state is ranked among the top-3 predictions over 96% of the time on average, underscoring the model’s effectiveness in prioritizing plausible next states.

**Table 3:** Link prediction performance of MambaGCN-SMI.

Protocol	AUC-ROC	AUC-PR	Recall@1	Recall@3	Recall@5
TCP	**0.985**	**0.951**	**0.968**	**0.985**	**0.991**
HTTP	0.964	0.903	0.886	0.942	0.962
SMTP	0.973	0.925	0.925	0.961	0.975
Modbus	0.976	0.938	0.956	0.977	0.984
DNP3	0.958	0.889	0.902	0.938	0.953
**Average**	0.971	0.921	0.927	0.961	0.973

In summary, under its optimal configuration, MambaGCN-SMI achieves strong performance across multiple evaluation perspectives. It predicts protocol behaviors with high accuracy (95.7%) and coverage (97.5%), while effectively recovering the structural characteristics of protocol state machines (node F1-score: 0.939, edge F1-score: 0.915). Link prediction metrics further confirm its robust ranking and discrimination ability, with average AUC-ROC of 0.971, AUC-PR of 0.921, and Recall@3 of 0.961. These results demonstrate that MambaGCN-SMI delivers superior and consistent inference performance on both public and private protocols.

#### Comparative analysis with existing methods.

To validate the superiority of MambaGCN-SMI, we compare it with three representative methods: PRETT [18], ReFSM [19], and Spita-PL [8]. It is important to note that these prior methods were primarily evaluated on classic protocol state machine inference benchmarks, namely FTP and SMTP, which have long been established as standard protocols for evaluating state machine inference algorithms due to their well-defined interaction logic and publicly available traces. To ensure a fair and meaningful comparison, our study also includes SMTP in the experimental dataset, alongside other protocols such as TCP, HTTP, Modbus, and DNP3. The performance comparison presented in Table 5 is specifically focused on these two common protocols.

However, we acknowledge that the source code for PRETT, ReFSM, and Spita-PL is not publicly available, making it infeasible to reproduce them under a unified experimental setup. Therefore, we directly cite their reported results from the original papers, where the evaluation settings—including datasets (FTP and SMTP), evaluation metrics (Accuracy and Coverage), and experimental conditions—are largely consistent with our setup. We have carefully verified that the SMTP data used in our study is comparable to those employed in the prior works. Nevertheless, minor differences in implementation details or traffic traces may exist, and we discuss this limitation in Section 5.2.

Table 4 presents the performance comparison between MambaGCN-SMI and existing methods on the FTP and SMTP protocols, which are classic benchmarks in protocol state machine inference. For the FTP protocol, MambaGCN-SMI achieves an Accuracy of 0.951 and a Coverage of 0.971, outperforming all compared methods. Compared to the best baseline Spita-PL (Accuracy: 0.942, Coverage: 0.978), our method improves Accuracy by 0.009 while maintaining comparable Coverage. For the SMTP protocol, the advantage of our method is more pronounced. MambaGCN-SMI achieves the highest Accuracy (0.962) and Coverage (0.981), surpassing Spita-PL by 0.041 in Accuracy and 0.027 in Coverage. This demonstrates its strong capability in modeling the more complex interaction patterns of SMTP.

**Table 4:** Performance comparison of state machine inference methods for different protocols.

Algorithm	PRETT	ReFSM	SpitaPL	MambaGCN-SMI
Accuracy (FTP/SMTP)	0.924/0.941	0.842/0.815	0.942/0.921	**0.951/0.962**
Coverage (FTP/SMTP)	0.949/0.963	0.863/0.884	**0.978**/0.954	0.971/**0.981**

*Note:* Best results are highlighted in bold. FTP and SMTP results are separated by a slash.

These results demonstrate that MambaGCN-SMI achieves superior and consistent performance across both classic protocol benchmarks, validating its effectiveness for protocol state machine inference.

### Ablation studies

To systematically verify the effectiveness of each core module within the MambaGCN-SMI framework, this section designs and conducts ablation experiments. Four comparative configurations are set up:

- Baseline Model: Removes both the Mamba encoder and the GCN simplification module, using raw bytes as state features and performing only direct threshold-based pruning;
- GCN-SMI: Removes only the Mamba encoder, retaining the GCN simplification module;
- Mamba-SMI: Removes only the GCN simplification module, retaining the Mamba encoder;
- Full MambaGCN-SMI.

**Fig 10:**
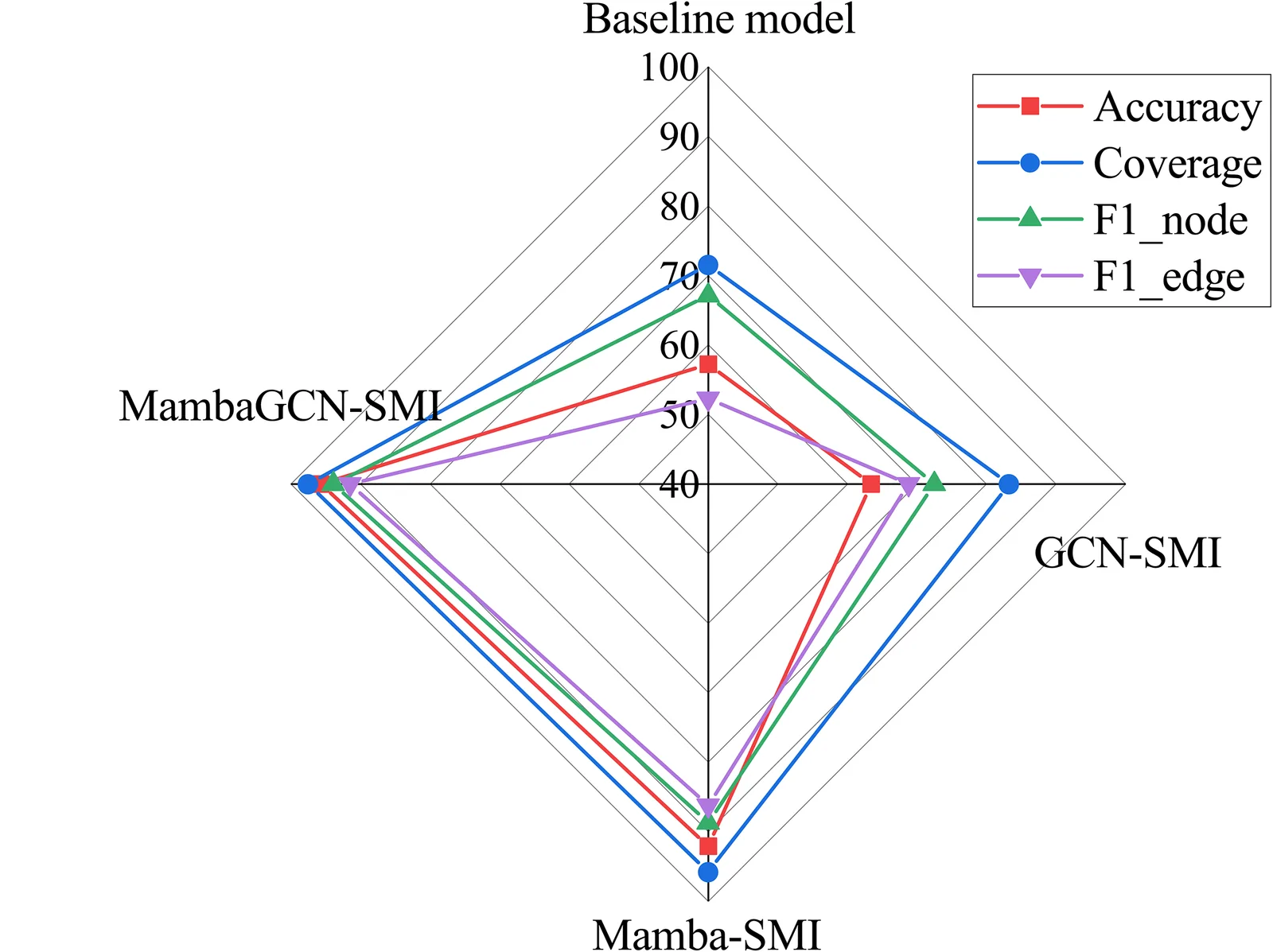
Performance comparison of the ablation experiment.

The experimental results are shown in Fig 10. Based on the ablation study results, the following conclusions can be drawn: (1) The GCN module enhances coverage and structural recovery: Adding the GCN simplification module to the baseline model increases Coverage and *F*1_edge_ by 11.7% and 16.5%, respectively. This demonstrates that GCN can effectively utilize the state transition topology to improve behavioral coverage and edge structure recovery. (2) The Mamba encoder is key to accuracy improvement: When using only the Mamba encoder, the Accuracy already reaches 92.1%, representing a 28.7% improvement over GCN-SMI. This proves that Mamba can significantly enhance the discriminative power of state features and is core to inference accuracy. (3) Comprehensive performance is significantly superior: The full model achieves the best performance across all four metrics, particularly showing a 39.2% improvement in *F*1_edge_ compared to the Baseline. This validates the effectiveness of the collaborative framework of “deep semantic modeling + graph structure optimization.”

In summary, Mamba and GCN play the roles of deep feature extraction and structural-semantic fusion, respectively, in protocol state machine inference. Their synergistic collaboration is essential for achieving high-precision, high-coverage, and high-structure-recovery inference performance.

### Class imbalance analysis

Protocol state machines inherently exhibit class imbalance, where certain states occur far more frequently than others. For example, in TCP sessions, the ESTABLISHED state appears much more often than transient states such as SYN-SENT or FIN-WAIT. This imbalance poses a challenge for state machine inference, as models may bias toward frequent states while neglecting rare but critical states. To assess the robustness of MambaGCN-SMI to class imbalance, we evaluate per-state performance by comparing F1-scores for high-frequency and low-frequency states.

Per-State F1-score Analysis: For each protocol, we rank states by their frequency and divide them into two groups: high-frequency states (top 50%) and low-frequency states (bottom 50%). We then compute the average node F1-score for each group. As shown in Table 5, the average F1-score for low-frequency states (0.921) is only slightly lower than that for high-frequency states (0.948), with a gap of just 0.027. This demonstrates that MambaGCN-SMI maintains strong performance even on rare states and does not simply bias toward frequent patterns.

**Table 5:** Per-State F1-score comparison between high-frequency and low-frequency states.

Protocol	High-Frequency States	Low-Frequency States	Gap
TCP	0.953	0.934	0.019
HTTP	0.892	0.871	0.021
SMTP	0.931	0.915	0.016
Modbus	0.962	0.947	0.015
DNP3	0.925	0.902	0.023
**Average**	**0.948**	**0.921**	**0.027**

*Note:* The gap represents the performance difference between high-frequency and low-frequency states.

Factors Contributing to Robustness Against Imbalance: The following design choices enable MambaGCN-SMI to handle class imbalance effectively:

1. State Transition Conditional Entropy: The clustering evaluation metric (Section 3.2) focuses on transition probabilities rather than raw state frequencies. By considering the dynamic behavior of the protocol rather than static state counts, this metric naturally reduces bias toward high-frequency states.
2. Graph Structure Learning: The GCN module leverages transition topology, allowing information to propagate from frequent to rare states through shared structural context. This enables the model to learn meaningful representations for rare states based on their relationships with frequent states.
3. Probabilistic Transition Modeling: By modeling state transitions as conditional probability distributions, our framework naturally accounts for inherent uncertainty and prevents overconfidence in frequent states.
4. Embedding-Based Representation: The Mamba encoder learns dense semantic embeddings for each state based on message content. Rare states with distinctive message patterns can still be well-separated in the embedding space, as their representations are driven by content rather than frequency alone.

It is worth noting that while the above analysis focuses on state-level performance, it also provides meaningful insight into transition-level behavior. Each state’s F1-score reflects the accuracy of all transitions originating from or arriving at that state, including those involving rare states. The strong results on low-frequency states (average F1-score 0.921) therefore imply that transitions associated with these states—including rare transitions—are also accurately inferred. This addresses potential concerns about model performance on low-frequency transitions, as the states that participate in such transitions are precisely those identified as low-frequency states.

These results confirm that MambaGCN-SMI effectively handles class imbalance and accurately infers both common and rare protocol behaviors. We acknowledge that extreme imbalance scenarios may require further specialized techniques, which we plan to explore in future work.

## Conclusions and future work

### Summary of work

This paper addresses the limitations in private protocol state machine inference by proposing MambaGCN-SMI, a deep learning-based protocol state machine inference method. The inference framework is constructed through the following three core modules: Firstly, a dual-level Mamba encoder is employed to extract protocol state features, effectively capturing long-range dependencies in protocol interactions through cascaded packet-level and session-level modeling. Secondly, an initial directed state transition graph is constructed based on clustering algorithms, achieving the mapping from continuous features to discrete structures. Finally, a GCN is utilized to learn topological embeddings of state nodes, enabling adaptive state simplification based on semantic similarity.

Experiments on both public and private protocol datasets demonstrate that MambaGCN-SMI outperforms existing methods in Accuracy (95.7%), Coverage (97.5%), and the state machine structural recovery metric F1-score, validating its effectiveness and generalization capability. Ablation studies further indicate that the Mamba encoder and the GCN simplification module play key roles in feature discriminability and structural optimization, respectively, and their synergy significantly enhances overall performance.

### Conclusions and future work

Although MambaGCN-SMI performs excellently across multiple metrics, as an exploratory study of deep learning in this domain, it still possesses the following limitations:

1. Strong Data Dependency: Model performance is influenced by the scale and distribution of training data; generalization ability may degrade in few-shot or atypical scenarios.
2. Insufficient Real-time Capability: Although Mamba offers linear complexity, stages like clustering and graph construction still affect overall inference efficiency, making it difficult to meet the demands of real-time analysis for high-speed traffic.
3. Weak Interpretability: The inferred state machine lacks explicit semantic annotation, which hinders intuitive understanding and result verification by analysts.
4. Class Imbalance: Protocol state distributions are inherently imbalanced. While our framework handles moderate imbalance well, extreme imbalance scenarios may still pose challenges.

### Future work directions

Building upon the research outcomes and limitations of MambaGCN-SMI, future work can focus on the following four core directions:

1. Extension and Adaptation for Complex Protocols: Investigate support mechanisms for various protocol types, such as encrypted protocols and lightweight Internet of Things (IoT) protocols, to enhance the method’s generality.
2. Improving Efficiency and Interpretability: Explore model lightweighting and online learning strategies to enhance real-time performance, and incorporate methods like attention mechanisms and external knowledge fusion to improve the interpretability of the inference process.
3. Inference Optimization in Few-Shot Environments: Leverage cross-protocol transfer learning techniques to reduce the model’s dependence on labeled data and sample size. Through continuous exploration in the aforementioned directions, it is anticipated that the applicability, robustness, and practical value of MambaGCN-SMI in real-world network environments can be further enhanced.
4. Handling Extreme Class Imbalance: Future work will explore specialized techniques for extremely rare states, including cost-sensitive learning, oversampling methods, focal loss, and meta-learning.

Through continuous exploration in the aforementioned directions, it is anticipated that the applicability, robustness, and practical value of MambaGCN-SMI in real-world network environments can be further enhanced.
